# Direct Electron Transfer of Hemoglobin on Manganese III Oxide-Ag Nanofibers Modified Glassy Carbon Electrode

**DOI:** 10.1155/2012/375831

**Published:** 2012-04-05

**Authors:** Masoud Negahdary, Gholamreza Mazaheri, Somyyeh Rad, Mohammadreza Hadi, Roya Malekzadeh, Mohammad Mahdi Saadatmand, Saeed Rezaei-Zarchi, Fariba Pishbin, Mojdeh Khosravian-hemami

**Affiliations:** ^1^Department of Biology, Payam-e-Noor University, Tehran 7371719578, Iran; ^2^Department of Biology, Shahrekord University, Shahrekord, Iran; ^3^Department of Biology, Payam-e-Noor University, Taft, Iran; ^4^Young Researchers Club, Islamic Azad University, Yazd Branch, Yazd, Iran

## Abstract

We investigated the electrochemical behavior of hemoglobin by glassy carbon electrode modified with Mn_2_O_3_-Ag nanofibers. The Mn_2_O_3_-Ag nanofibers were used as facilitator electron transfer between Hb and glassy-carbon-modified electrode. The Mn_2_O_3_-Ag nanofibers are studied by scanning electron microscopy (SEM) and transmission electron microscopy (TEM). The hemoglobin showed a quasireversible electrochemical redox behavior with a formal potential of −49 mV (versus Ag/AgCl) in 0.1 M potassium phosphate buffer solution at pH 7.0. The designed biosensor possesses good stability and reproducibility and achieves 95% of the steady-state current in less than five seconds.

## 1. Introduction

Hemoglobin is a remarkable molecular machine that uses motion and small structural changes to regulate its action. Oxygen binding at the four heme sites in hemoglobin does not happen simultaneously. Once the first heme binds oxygen, it introduces small changes in the structure of the corresponding protein chain. The main function of red blood cell is transfer of O_2_ from lungs to tissue and transfer of CO_2_ from tissue to lungs. To accomplish these functions, red cells have hemoglobin (Hb). The Hb has iron in center of its structure [1]. The iron has important role in Hb structure [2]. We too used Hb in this project. Glassy carbon derives its name from exhibiting fracture behavior similar to glass, from having a disordered structure over large dimensions (although it contains a graphitic microcrystalline structure), and because it is a hard shiny material, capable of high polish [3–5]. Glassy carbon is particularly useful in electrochemical applications because of its low electrical resistivity, impermeability to gases, high chemical resistance, and because it has the widest potential range observed for carbon electrode. Glassy carbon was first prepared by Yamada and Sato in 1962 by the hight emperature pyrolysis of phenolic resin, and later by Davison who used cellulose as the starting material [6, 7]. They concluded that glassy carbon consists of long microfibrils that twist, bend, and interlock to form interfibrillar bonds, and that these microfibrils are randomly oriented [8]. Because of the desire to impart selectivity to electrochemical reactions and to control electron transfer kinetics, several investigators have utilized adsorption or covalent bonding of catalysts to the glassy carbon surface. The intent of these modifications is to control the interaction of molecules and ions with the electrode surface [9]. Small fibers in the submicron range, in comparison with larger ones, are well known to provide better filter efficiency at the same pressure drop in the interception and inertial impaction regimes [10, 11]. Previous researches shown such effect by assuming nonslip flow at fiber surface [12]. While smaller fiber size leads to higher-pressure drop, interception and inertial impaction efficiencies will increase faster, more than compensating for the pressure drop increase. Thus, in the particle size of interest, that is, from submicron and up, better filter efficiency can be achieved at the same pressure drop, or conversely, the same filter efficiency at lower pressure drop can be achieved with smaller fiber sizes. Polymeric nanofibers can be made using the electrospinning process, which has been described in the literature [13] and in patents [14]. Electrospinning uses an electric field to draw a polymer solution from the tip of a capillary to a collector. A voltage is applied to the polymer solution, which causes a jet of the solution to be drawn toward a grounded collector. The fine jets dry to form polymeric fibers, which can be collected on a web. The electrospinning process has been documented using a variety of fiber-forming polymers [15, 16]. By choosing a suitable polymer and solvent system, nanofibers with diameters in the range of 40–2000 nm can be made. PVP is a kind of polymers which is able to adsorb through many points of the molecule on the surface of steel, copper, and zinc and reduce the corrosion rate of the material [17–19]. PVPs are considered as food additives and, hence, their addition will not raise health concerns for treating RO water. However, the effect of the molecular weight of polyvinylpyrrolidone, its concentration, and temperature on the corrosion properties of 316 L stainless steel in reverse osmosis water were not evaluated.

## 2. Material and Methods

### 2.1. Electrospinning Nanofibers

Electrospinning is a process of applying a high-voltage electric field (several to tens of kilovolts) to generate electrically charged jets from polymer solutions or melts and further to produce polymer (nano) fibers. This technique is quite similar with the commercial process for drawing microscale fibers; however, it is more suitable for generating nanofibers, because the elongation can be accomplished by a contactless scheme through the application of an external electric field [20]. Generally, generation of porous surface on a bulk electrospinning nanofiber can be realized through two different ways. The first one is based on the selective removal of a component from nanofibers made of a composite or blend material, while the other one involved the use of phase separation of different polymers during electrospinning under the application of proper sinning parameters [16]. Both the pore size and the density are controllable by changing the parameters. For instance, in PLA/PVP electrospinning nanofibers, more porosity can be generated when the two materials are loaded in equal amounts comparing to the 11 corresponding products by different proportion of PLA/PVP. It can be attributed to the rapid-phase separation and solidification in the spinning jet [14–17]. PLA is contraction name of polylactic acid, and it is a thermoplastic polymer made from lactic acid and has mainly been used for biodegradable products, such as plastic bags and planting cups [18]. The PLA/PVP/nanofibers have been prepared by electrospinning [19]. It was found that the average diameter of the PLA/PVP/nanofibers lead fibers became larger, and the morphology of the fibers became finer with the content of PLA increasing. The formation of pores is also affected by the solvent vapor pressure and the humidity in atmosphere. The cooling effect that comes from rapid evaporation of a highly volatile solvent might induce the polymers to separate into different phases in liquid jet. Because of evaporative cooling and condensation, water droplets could also be formed within the fibers to promote the formation of porous nanofibers [20–22]. The electrospun nanofibers exhibit several unique features, which enable the prevalent utilization of them. Because electrospinning is a continuous process without any contact force for elongation, the fibers can be as long as several kilometers and can be further assembled into a 3D mat with porous structure. At the same time, electrospun fibers can have a thinner diameter and surface-to-volume ratio, due to the presence of porous structure. In addition, due to the simple fabrication process and the diversity of suitable materials, the electrospinning technique and its resultant nanofiber product have attracted increasing attention. These properties potentiate the use of the electrospun nanofibers in various applications such as reinforced composites, nanofiber-based membranes, nanofiber-based support for enzyme, and catalyst [23–25].

### 2.2. Reagents

Hb, manganese(II) nitrate tetrahydrate, and poly(vinylpyrrolidone) (PVP, MW = 1,300,000) were purchased from Sigma. Silver nitrate and other materials were supplied by Merck. 0.1 M phosphate buffer solutions with various pH values were prepared by mixing stock standard solutions of Na_2_HPO_4_ and NaH_2_PO_4_ and adjusting the pH values with NaOH and H_3_PO_4_ solution. All solutions used in the experiments were prepared with deionized water generated by a Barnstead water system.

#### 2.2.1. Preparation of Mn_**2**_O_**3**_-Ag Nanofibers

44 wt% Mn(NO_3_)_2_, 11 wt% AgNO_3_, and 44 wt% PVP were dissolved in DMF. The solution was kept under magnetic stirring for 2 h and then loaded into a plastic syringe equipped with a 23-gauge needle made of stainless steel. Electrospinning process was conducted at an applied voltage of 20 kV with a feeding rate of 0.3 mL/h and a collection distance of 15 cm. The nanofibers were collected on aluminum foil and then calcined under air atmosphere at 500°C for 3 h for the degradation of PVP and the decomposition of Mn(NO_3_)_2_ and AgNO_3_:

(1) $$\begin{array}{c} \text{4Mn}\left(\text{N}{\text{O}}_{\text{3}}\right)\overset{∆}{\to }\text{2M}{\text{n}}_{\text{2}}{\text{O}}_{\text{3}}+\text{8N}{\text{O}}_{\text{2}}↑+\;{\text{O}}_{\text{2}}↑\\ \text{2AgN}{\text{O}}_{\text{3}}\overset{∆}{\to }\text{2Ag}+2\text{N}{\text{O}}_{\text{2}}↑+\;{\text{O}}_{\text{2}}↑. \end{array}$$

Electrospinning is a process of applying a high-voltage electric field (several to tens of kilovolts) to generate electrically charged jets from polymer solutions or melts and further to produce polymer (nano) fibers. So, in this study, we used from this method to produce Mn_2_O_3_-Ag nanofibers.

#### 2.2.2. Preparation of Mn_**2**_O_**3**_-Ag-Nanofibers-Modified Glassy Carbon Electrode

The most commonly used carbon-based electrode in the analytical laboratory is glassy carbon (GC). It is made by pyrolyzing a carbon polymer, under carefully controlled conditions, to a high temperature like 2000°C. An intertwining ribbon-like material results with retention of high conductivity, hardness, and inertness. Glassy carbon electrode (GCE, dia. 3 mm) was polished with 1 *μ*m and 0.05 *μ*m alumina slurries sequentially and then rinsed with DI water. After that, the electrode was sonicated in deionized water and finally dried under ambient conditions. To prepare the modified GCE, The Mn_2_O_3_-Ag nanofibers/glassy carbon electrode was placed into a fresh PBS including 5 mg mL^−1^ Hb (pH 7.0, 3 to 5°C) for 8 h. At the end, the modified electrode was washed in deionized water and placed in PBS (PH 7.0) at a refrigerator (3 to 5°C), before being employed in the electrochemical measurements as the working electrode.

#### 2.2.3. Apparatus and Electrochemical Measurement

A JEOL 6335F field-emission scanning electron microscope (SEM) was used to examine the morphology and the size of the as-prepared nanofibers. More detailed morphology and selected area electron diffraction (SAED) patterns of Mn_2_O_3_-Ag nanofibers were obtained with a Tecnai T12 transmission electron microscope (TEM) operated at 120 kV. XRD pattern was obtained with Oxford diffraction XcaliburTM PX Ultra with ONYX detector to study the crystal structure of Mn_2_O_3_-Ag nanofibers measurements which were carried out with a potentiostat/galvanostat (Model 263A, EG&G, USA) using a single compartment voltammetric cell, equipped with a platinum rod auxiliary electrode. All experiments were conducted using a three-electrode electrochemical cell (10 mL volume with a working volume of 5 mL), with a working electrode, an Ag/AgCl reference electrode, and a platinum wire counter electrode. Electrochemical measurements were carried out with a potentiostat/galvanostat (Model 263A, EG&G, USA) using a single compartment voltammetric cell, equipped with a platinum rod auxiliary electrode.

## 3. Results and Discussion

### 3.1. Characterization of Mn_**2**_O_**3**_-Ag Nanofibers

**Figure 1:**
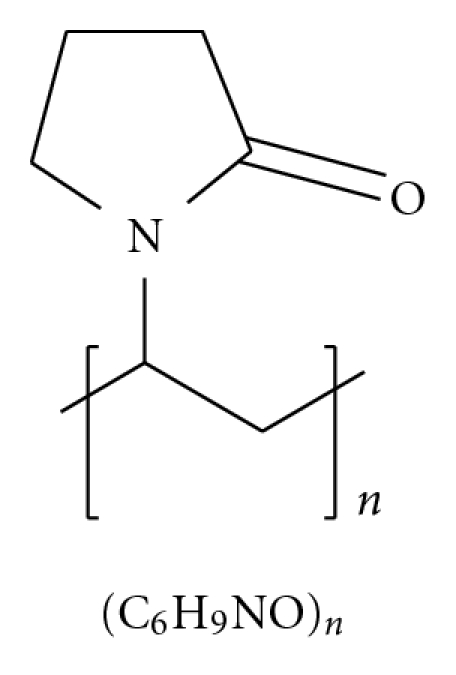
Molecular structure of polyvinylpyrrolidone (PVP).

**Figure 2:**
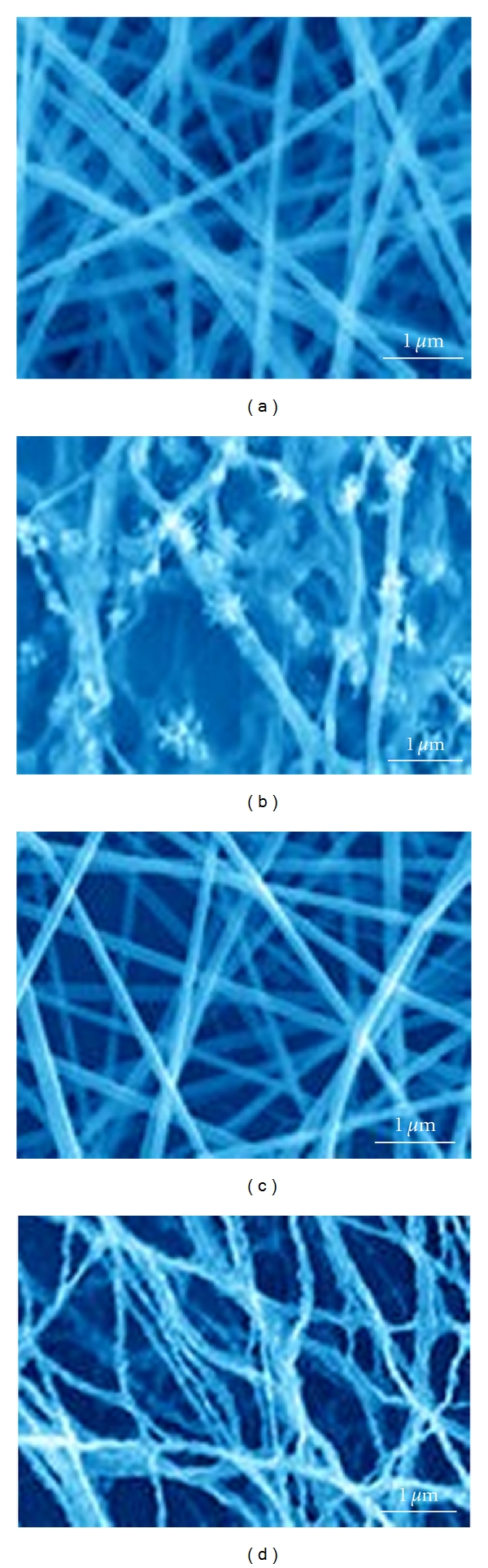
SEM images of (a) PVP-Mn(NO_3_)_2_-AgNO_3_ nanofibers, (b) Mn_2_O_3_-Ag nanofibers, (c) PVP-Mn(NO_3_)_2_ nanofibers, and (d) Mn_2_O_3_ nanofibers, respectively.

SEM was first employed to investigate the morphology of the Mn_2_O_3_-Ag nanofibers.  Figure 2(a) presents a typical SEM image of electrospun precursory PVP-Mn(NO_3_)_2_-AgNO_3_ nanofibers. PVP was used for characterization of Mn_2_O_3_-Ag nanofibers, because PVP binds to polar molecules exceptionally well, owing to its polarity. This has led to its application in coatings for increase of photo quality in microscopic studies. The molecular structure of PVP can be observed in Figure 1. PVP is soluble in water and other polar solvents. When dry, it is a light flaky powder, which readily absorbs up to 40% of its weight in atmospheric water. In solution, it has excellent wetting properties and readily forms films. This makes it good as a coating or an additive to coatings. PVP is a branched polymer, that is, its structure is more complicated than linear polymer, but it too is in a two-dimensional plane. Moreover, because Mn_2_O_3_-Ag nanofibers have polar groups too, a good bind was created between this nanofiber and PVP.

 After calcination, the as-prepared Mn_2_O_3_-Ag composite nanofibers (Figure 2(b)) exhibit a porous network structure, and their surfaces are no longer as smooth as the precursory nanofibers. Such feature endows the nanofibers with high surface-to-volume ratio, which could provide not only a large surface area for Hb loading but also a large interface for direct electron transfer of Hb. As a comparison, the nanofibers prepared by single metal salt (Mn(NO_3_)_2_) with PVP and its calcined product (Mn_2_O nanofibers) are presented in Figures 2(c) and 2(d), respectively.

### 3.2. XRD Diffraction of Mn_**2**_O_**3**_-Ag Nanofibers

**Figure 3:**
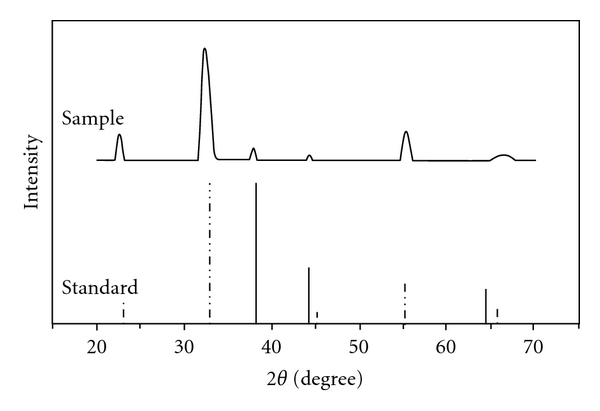
XRD patterns for the standard values of JCPDS 41-1442 (Mn_2_O_3_, dashed line), JCPDS 04-0783 (Ag, solid line), and the asprepared porous Mn_2_O_3_-Ag nanofibers.

The composition and crystal structure were characterized by XRD (Figure 3). The XRD spectrum of Mn_2_O_3_-Ag nanofibers matches the combination of the standard spectrum of JCPDS 41-1442 (Mn_2_O_3_) and JCPDS 04-0783 (Ag). The formation of face-centered cubic crystalline Mn_2_O_3_ is revealed by the diffraction peaks at *2θ* values of 32.951, 38.234, 45.178, 49.347, 55.189, and 65.806, corresponding to (111), (200), (220), (311), (222), and (400) crystal planes, respectively, while the diffraction peaks at *2θ* values of 38.116, 44.277, and 64.426, which correspond to (111), (200), and (220) crystal planes, respectively, indicate the formation of cubic crystalline Ag.

#### 3.2.1. Direct Electrochemistry of Hb/Mn_**2**_O_**3**_-Ag Nanofibers/GCE

**Figure 4:**
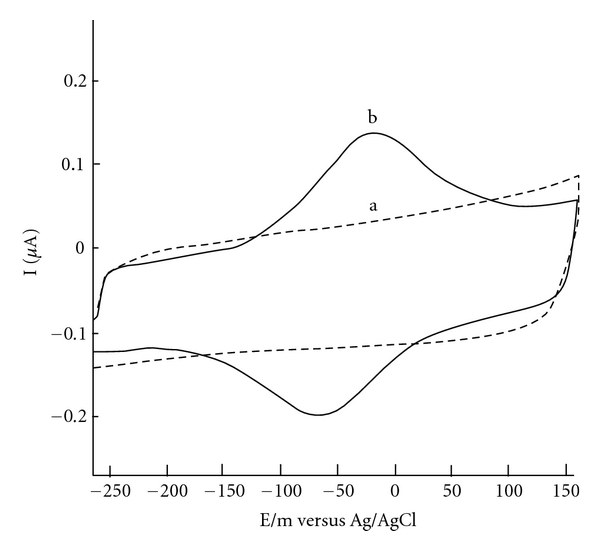
Cyclic voltammograms, using (a) bare glassy carbon electrode in 0.1 M phosphate buffer solution and (b) Hb/Mn_2_O_3_-Ag nanofibers/glassy carbon electrode in 0.1 M phosphate buffer solution (scanrate: 100 mV s^−1^).

Cyclic voltammetry (CV) was used to characterize the modification of electrodes. The cyclic voltammograms (CVs) of different modified electrodes were obtained in 0.1 M PBS with pH 7.0. No redox peak is observed for the CVs of bare GC electrode. Compared with bare GC electrode, the background current of Mn_2_O_3_-Ag-nanofibers-modified electrode is apparently larger, which indicates that the effective electrode surface area is significantly enhanced by use of Mn_2_O_3_-Ag nanofibers to modify the electrode. However, the CVs of Hb/Mn_2_O_3_-Ag nanofibers/GC electrode give a pair of well-defined redox peaks at −75 mV at scan rate of 100 mV/s, characteristic of heme Fe(III)/Fe(II) redox couples of Hb, suggesting that direct electron transfer has been achieved between Hb and modified electrode. The electron transfer of the proteins, at the bare electrodes is very slow so that the redox peak of proteins can usually be observed.  Figure 4(a) shows a cyclic voltammogram (CV) of the bare glassy carbon electrode.  Figure 4(b) shows a cyclic voltammogram of Hb/Mn_2_O_3_-Ag nanofibers/glassy carbon electrode in 0.1 M phosphate buffer at pH 7.0. The Hb showed quasireversible electrochemical behavior with a formal potential of −49 Mv (versus Ag/AgCl); cathodic and anodic peaks were not observed using the bare graphite electrode. This shows that Mn_2_O_3_-Ag nanofibers act as a facilitator of electron transfer from the redox species of Hb to the electrode surface and vice versa. These results are in line with the previous work that explains the behavior of nanoparticles as the facilitators of electron transfer.

**Figure 5:**
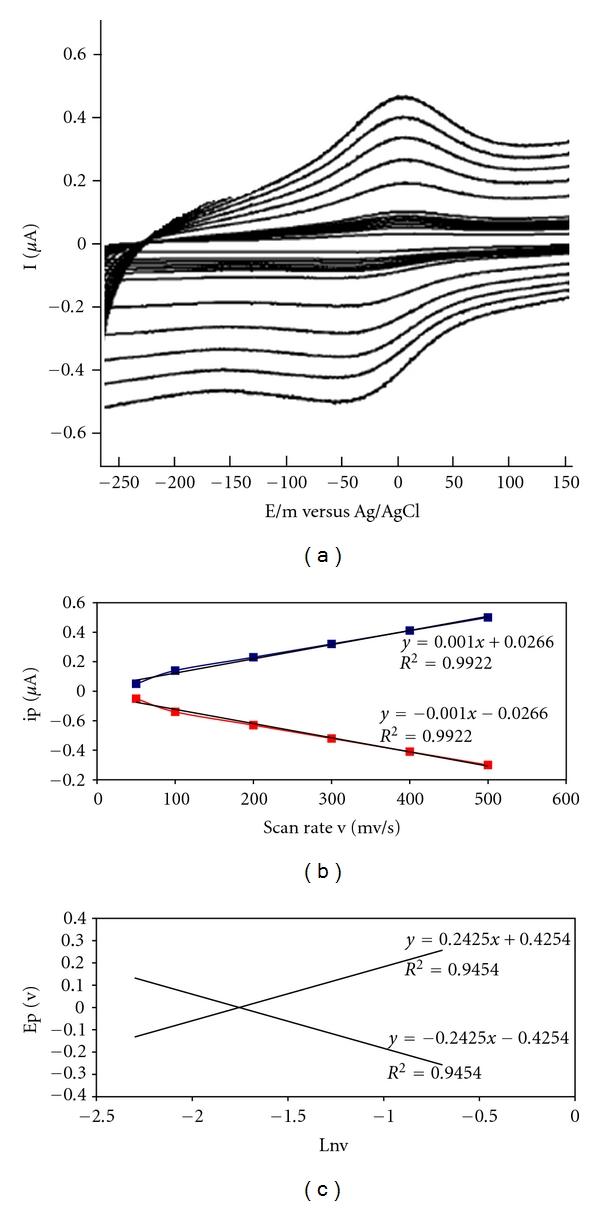
(a) Typical cyclic voltammograms of Hb/Mn_2_O_3_-Ag nanofibers/glassy carbon electrode at different scan rates. The voltammograms (from inner to outer) designate scan rates of 50, 100, 200, 300, 400, and 500 mV s^−1^, respectively. (b) Dependence of the anodic and cathodic peak currents on the scan rates the red line is cathodic and the blue line is anodic. (c) Relationship between the peak potential (*E*_p_) and the natural logarithm of scan rate (ln⁡*v*) for Hb/Mn_2_O_3_-Ag nanofibers/glassy carbon electrode. All the data were obtained at pH 7.0 and in 0.1 M phosphate buffer solution.

In Figure 4(a), one sees cyclic voltammogram for bare and modified glassy carbon electrode in 50, 100, 200, 300, 400, and 500 scan rates. These data showed that the Mn_2_O_3_-Ag nanofibers were successfully assembled on the glassy carbon electrode surface. The redox peak currents increase linearly with the square root of the scan rate. With an increasing scan rate, the anodic peak potential of adsorbed Hb shifted to a more positive value, while the cathodic peak current shifted in a negative direction. The redox peak currents were proportional to the scan rate (Figure 5); thus, the electrode reaction was typical of the surface-controlled quasireversible process. One can see in Figure 5(b) the dependence of the anodic and cathodic peak currents on the scan rates that the red line is cathodic and the blue line is anodic. The kinetics of the heterogeneous electron transfer was analyzed using the model of Laviron [9]. The plot of cathodic peak potentials versus the logarithm of scan rates gave a charge transfer coefficient *α* of 0.52. For scan rates from 50 to 500 mV/s, the electron transfer rate constant *k*_*s*_ was estimated to be (1.9) s^−1^. These statements discussed below indicate that Hb is strongly adsorbed onto the surface of modified electrode. As could be seen in Figure 5(c), in the range from 200 to 500 mV s^−1^, the catholic peak potential (*E*_pc_) changed linearly versus ln⁡*v* with a linear regression equation of *y* = −0.2425*x* − 0.4254, *R* ^2^ = 0.945, according to the following equation [26–28],

(2) $$E_{\text{p}}=E^{{}^{\circ}\prime }+\frac{RT}{\alpha nF}-\frac{RT}{\alpha nF}\ln v^{\prime },$$

where *α* is the cathodic electron transfer coefficient, *n* is the number of electrons, *R*, *T*, and *F* are gas, temperature, and Faraday constant, respectively (*R* = 8.314 J mol^−1 ^K^−1^, *F* = 96493 C/mol, *T* = 298 K), and *αn* is calculated to be 0.52. Given 0.3 < *α* < 0.7 in general [9], it could be concluded that *n* = 1 and *α* = 0.52. From the width of the peak at midheight and low scan rate, we can also obtain *n* = 1 [9]. Therefore, the redox reaction between Hb and modified graphite electrode is a single electron transfer process. In order to calculate the value of apparent heterogeneous electron transfer rate constant (*k*_*s*_), the following equation was used [26–28]:

(3) $$\begin{array}{cc} \log k_{s} & =\alpha \log\left(1-\alpha \right)+\left(1-\alpha \right)\log\alpha -\log\left(\frac{RT}{nFv}\right)\\ & \;-\alpha \left(1-\alpha \right)\frac{nF\Delta E_{\text{p}}}{2.3RT}. \end{array}$$

The *k*_*s*_ was calculated to be 1.90 s^−1^. Moreover, this *k*_*s*_ indicates that transfer of electron is good and fast in Hb-modified glassy carbon electrode.

#### 3.2.2. Design a Hydrogen Peroxide Biosensor

The designed biosensor is introduced as a hydrogen biosensor because it has oxidation and reduction reactions in Hb; when Fe^2+^ changes to Fe^3+^, the oxidation reaction occurred, and when Fe^3+^ changes to Fe^2+^, the reduction reaction occurred. The reduction state is an important matter for designing hydrogen peroxide biosensors [29].

**Figure 6:**
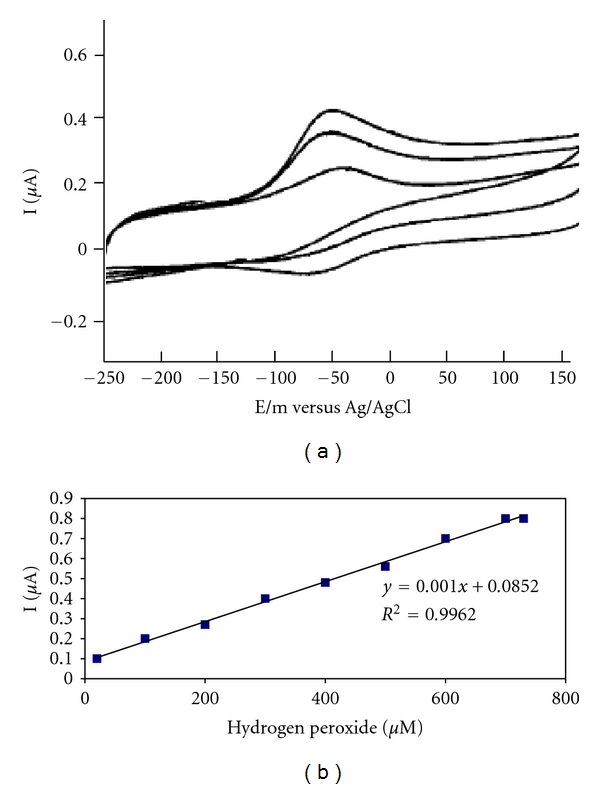
(a) Cyclic voltammograms obtained at an Hb/Mn_2_O_3_-Ag nanofibers/glassy carbon electrode in 50 *μ*M phosphate buffer solution (pH 7.0) for different concentrations and (b) the relationship between cathodic peak current of Hb and different concentrations of H_2_O_2_ (scan rate: 100 mV s^−1^).

The electrocatalytic reactivity of Hb/Mn_2_O_3_-Ag nanofibers/GC electrode toward H_2_O_2_ was investigated by CV.  Figure 6 displays the cyclic voltammograms obtained for the hydrogen peroxide biosensor in PBS (pH 7.0) containing varied concentration of H_2_O_2_ in the absence of oxygen. The catalytic reduction of hydrogen peroxide at the biosensor can be seen clearly in Figure 5. With the addition of H_2_O_2_, the reduction peak current increases obviously, while the oxidation peak current decreases (Figures 6(a) and 6(b)), indicating a typical electrocatalytic reduction process of H_2_O_2_. However, no similar cathodic peak corresponding to the reduction of H_2_O_2_ can be observed at bare GC, Mn_2_O_3_-Ag nanofibers/GC electrode under the same condition, so it can be concluded that Hb immobilized on Mn_2_O_3_-Ag nanofibers/GC electrode shows good catalytic activity toward hydrogen peroxide [19].  Figure 6 shows the sensor response. The sensor was found to have sensitivity in the range of 20 to 700 *μ*M based upon the mean of the slope found from the points on the response curve. As can be observed, the sensor response shows good linearity in this range. The correlation factor, *R* ^2^, was found to be 0.9962.

### 3.3. Influence of pH and Applied Potential on Biosensor Response

**Figure 7:**
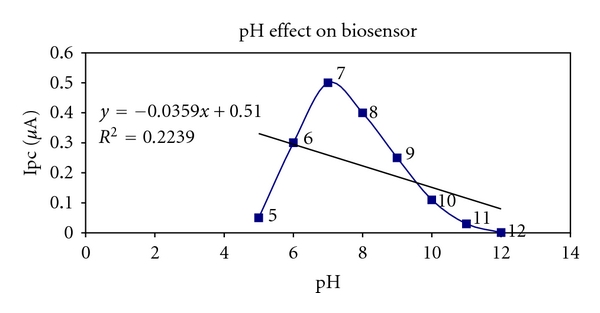
Dependence of the current response of Hb/Mn_2_O_3_-Ag nanofibers/GC electrode to 50.0 *μ*M H_2_O_2_ on the pH of buffer solutions.

In order to obtain an efficient biosensor for H_2_O_2_, the influence of pH and applied potential on the response of Hb/Mn_2_O_3_-Ag nanofibers/GC electrode were investigated. The change of chronoamperometric current with the pH under constant hydrogen peroxide concentration (50.0 *μ*M) is shown in Figure 7. As can be seen, the maximum response appears at pH 7.0. So the buffer solution of pH 7.0 was selected for experiments. This designed biosensor possesses good stability and reproducibility and achieves 95% of the steady-state current in less than 5 s.

## 4. Conclusion

A new biosensor for H_2_O_2_ was prepared based on Hb/Mn_2_O_3_-Ag nanofibers/glassy carbon electrode. Hb retained well in Mn_2_O_3_-Ag nanofibers/GCE, which combined the utilities of Mn_2_O_3_-Ag nanofibers facilitating the electron transfer. At Hb/Mn_2_O_3_-Ag nanofibers/GCE, the cyclic voltammogram exhibits a pair of redox peaks corresponding to a surface-controlled electrode process with a single-proton transfer. The designed biosensor displays a high affinity and sensitivity to H_2_O_2_. The sensor shows good reproducibility and stability.

## References

[1] Davis F, Higson SPJ (2007). Biofuel cells-recent advances and applications. *Biosensors and Bioelectronics*.

[2] Potter MC (1912). Electrical effects accompanying the decomposition of organic compounds. *Proceedings of the Royal Society*.

[3] Yahiro AT, Lee SM, Kimble DO (1964). Bioelectrochemistry. I. Enzyme utilizing bio-fuel cell studies. *Biochimica et Biophysica Acta*.

[4] Sulak MT, Gökdoğan O, Gülce A, Gülce H (2006). Amperometric glucose biosensor based on gold-deposited polyvinylferrocene film on Pt electrode. *Biosensors and Bioelectronics*.

[5] Bullen RA, Arnot TC, Lakeman JB, Walsh FC (2006). Biofuel cells and their development. *Biosensors and Bioelectronics*.

[6] Lim KG, Palmore GTR (2007). Microfluidic biofuel cells: the influence of electrode diffusion layer on performance. *Biosensors and Bioelectronics*.

[7] Heller A (2004). Miniature biofuel cells. *Physical Chemistry Chemical Physics*.

[8] Kamitaka Y, Tsujimura S, Setoyama N, Kajino T, Kano K (2007). Fructose/dioxygen biofuel cell based on direct electron transfer-type bioelectrocatalysis. *Physical Chemistry Chemical Physics*.

[9] Rezaei-Zarchi S, Negahdary M, Doroudian M (2011). Direct electron transfer of Myoglobin on nickel oxide Nanoparticles modified graphite electrode. *Advances in Environmental Biology*.

[10] Wang J, Musameh M (2003). Carbon nanotube/Teflon composite electrochemical sensors and biosensors. *Analytical Chemistry*.

[11] Wu K, Fei J, Hu S (2003). Simultaneous determination of dopamine and serotonin on a glassy carbon electrode coated with a film of carbon nanotubes. *Analytical Biochemistry*.

[12] Zhang X-H, Wang S-F (2005). Determination of ethamsylate in the presence of catecholamines using 4-amino-2-mercaptopyrimidine self-assembled monolayer gold electrode. *Sensors and Actuators, B*.

[13] Wang JX, Sun XW, Wei A (2006). Zinc oxide nanocomb biosensor for glucose detection. *Applied Physics Letters*.

[14] Ding Y, Wang Y, Su L, Bellagamba M, Zhang H, Lei Y (2010). Electrospun Co_3_O_4_ nanofibers for sensitive and selective glucose detection. *Biosensors and Bioelectronics*.

[15] Rubio Retama J, López Cabarcos E, Mecerreyes D, López-Ruiz B (2004). Design of an amperometric biosensor using polypyrrole-microgel composites containing glucose oxidase. *Biosensors and Bioelectronics*.

[16] Lu X, Zhou J, Lu W, Liu Q, Li J (2008). Carbon nanofiber-based composites for the construction of mediator-free biosensors. *Biosensors and Bioelectronics*.

[17] Amalraj AJ, Sundaravadivelm M, Regis APP, Rajendran S Corrosion inhibition by polyvinylpyrrolidone.

[18] Gürten AA, Erbil M, Kayakirilmaz K (2005). Effect of polyvinylpyrrolidone on the corrosion resistance of steel. *Cement and Concrete Composites*.

[19] Jianguo Y, Lin W, Otieno-Alego V, Schweinsberg DP (1995). Polyvinylpyrrolidone and polyethylenimine as inhibitors for the corrosion of a low carbon steel in phosphoric acid. *Corrosion Science*.

[20] Oksman K, Skrifvars M, Selin JF (2003). Natural fibres as reinforcement in polylactic acid (PLA) composites. *Composites Science and Technology*.

[21] Xu J, Zhang J, Gao W, Liang H, Wang H, Li J (2009). Preparation of chitosan/PLA blend micro/nanofibers by electrospinning. *Materials Letters*.

[22] Ebbesen TW, Ajayan PM (1992). Large-scale synthesis of carbon nanotubes. *Nature*.

[23] Vassilyev YB, Khazova OA, Nikolaeva NN (1985). Kinetics and mechanism of glucose electrooxidation on different electrode-catalysts. Part II. Effect of the nature of the electrode and the electrooxidation mechanism. *Journal of Electroanalytical Chemistry*.

[24] Gorman A, McGowan A, Cotter TG (1997). Role of peroxide and superoxide anion during tumour cell apoptosis. *FEBS Letters*.

[25] Zhuang Z, Su X, Yuan H, Sun Q, Xiao D, Choi MMF (2008). An improved sensitivity non-enzymatic glucose sensor based on a CuO nanowire modified Cu electrode. *Analyst*.

[26] Bard AJ, Faulkner LR (2001). Electrochemical methods. *Fundamentals and Applications*.

[27] Laviron E (1979). General expression of the linear potential sweep voltammogram in the case of diffusionless electrochemical systems. *Journal of Electroanalytical Chemistry*.

[28] Laviron E (1979). The use of linear potential sweep voltammetry and of a. c. voltammetry for the study of the surface electrochemical reaction of strongly adsorbed systems and of redox modified electrodes. *Journal of Electroanalytical Chemistry*.

[29] Banerjee R, Cassoly R (1969). Preparation and properties of the isolated *α* and *β* chains of human hemoglobin in the ferri state. Investigation of oxidation-reduction equilibria. *Journal of Molecular Biology*.

